# A conversation with Peter Hotez

**DOI:** 10.1172/JCI176284

**Published:** 2023-11-01

**Authors:** Ushma S. Neill

A committed champion of vaccines and vaccine diplomacy, Dr. Peter Hotez is the Dean of the National School of Tropical Medicine and Professor of Pediatrics and Molecular Virology and Microbiology at the Baylor College of Medicine. As codirector of the Texas Children’s Hospital Center for Vaccine Development, Hotez (Figure 1) has led the development and clinical trials of low-cost vaccines for hookworm infection, schistosomiasis, leishmaniasis, Chagas disease, and various coronaviruses. His persistence in the face of online and in-person harassment is impressive. This condensed transcript is accompanied by an interview with Dr. Hotez from a stop on his book tour at Memorial Sloan Kettering Cancer Center on September 20, 2023; see the video for a discussion about issues pertaining to the rise of antiscience at www.jci.org/videos/cgms

*JCI*: Can you start by telling us about your family and early influences?

Hotez: I grew up in West Hartford, Connecticut, and ever since I was a kid, I wanted to be a scientist investigating microbes. I had a microscope as a kid, and I used to go down and collect pond water and look at microorganisms under a microscope; I also had an early fascination with maps. I was a nerdy kid who loved maps and microbes and combining that together, and I guess you get tropical medicine and infectious diseases.

My father worked at United Technologies and taught business at the local community college, and my mother stayed home with my three siblings and me. I had a great-uncle, David Krech, who was an important psychologist and social justice activist at UC Berkeley, and my uncle, Irving Goldberg, who was a prominent biomedical scientist and Harvard professor. Another main role model for me was one of my cousins who was a Holocaust survivor. Phil Lazowski — he was a rabbi and early on he taught me the concept of tikkun olam (repairing the world) and about our obligation to repair the world; I thought being a physician and a scientist would embody that ideal.

*JCI*: What was your path to Yale for your undergrad degree in molecular physics and biochemistry?

Hotez: At the time, there were two professors that were starting to apply modern technology to the study of parasites. This was shortly after the first gene was cloned in the mid 1970s, and Frank Richards and Curtis Patton were two professors at Yale who were starting to mechanistically study African sleeping sickness and cloning genes from trypanosomes. That’s what I wanted to do and that’s why I went to Yale to work in those laboratories.

*JCI*: You were that committed even in high school?

Hotez: Even then! I knew I wanted to do molecular parasitology since I was very young.

Then I was an MD-PhD student in New York, at Rockefeller and Weill Cornell and Sloan Kettering; I was taken by the impact of vaccines and decided that I wanted not only to study parasites, but also to see if it was possible to apply modern molecular biology technology to the study of complicated helminths or parasites toward making vaccines. Anthony Cerami was a professor at Rockefeller who invited me into his lab, and I worked on a hookworm vaccine for my PhD thesis. And now, 40 years later, that vaccine is in phase 2 clinical trials.

*JCI*: You landed in pediatric infectious disease after a pediatrics residency at Mass General and clinical fellowship at Yale. Did you ever think that you were going to be a dedicated clinician?

Hotez: I wanted to do some clinical work, but didn’t want to make it my major activity. But what clinical specialty is most closely allied with vaccines? Pediatrics. When I went back to Yale, I went back to the same lab I worked in as an undergraduate (Frank Richards). I did well, and back then the new investigator R01 awards were called R29s; I’d gotten an R29 and found a space to clear out to create a small lab. I loved it.

I was at Yale for a decade and rose to the rank of associate professor. Then I had the opportunity to head a basic science department (Department of Microbiology) at George Washington University. I had wanted to be in DC to apply our discoveries to actually making vaccines. For neglected tropical diseases, there’s no obvious path. The classical model is, you make a discovery, you license it to a pharma company, and then they run with it to make it into a usable technology. For something like a hookworm vaccine, there is no pharma company interest given the lack of a lucrative US-based market. I wanted to see if we could create a model by which you take it as far as you can in an academic health center. George Washington gave me that opportunity, but the other part was — where do you learn how to actually produce vaccines at scale? You can’t walk into Merck or Pfizer and say, “show me how to make a vaccine.” Walter Reed Army Institute of Research (WRAIR) was up the road, and that is one of the few places where vaccines were being made in the nonprofit sector. Former scientists at WRAIR taught us how to make the transition from being a pure academic laboratory making discoveries into doing all that was needed to make a vaccine: process development, quality control, quality assurance, and so much more.

*JCI*: I note from your CV that this is also the time when you started engaging with the government — and foreign governments — more frequently, and you started to write more pieces in the popular press.

Hotez: Well, being in Washington, DC, at that time was akin to Rome at the height of the Roman Empire. So when in Rome! There were amazing policy opportunities. One of the things I realized was that if you are a subject matter expert and a professor and you’re passionate about what you do, people will listen. This was my first attempt at public engagement — back in about 2000, it was the launch of the Millennium Development Goals; one of the goals was specifically around infectious diseases. It was to combat HIV/AIDS, malaria, and other diseases.

I realized everything I’d devoted my life to was being called “other diseases,” and from an advocacy perspective, that was a nonstarter. I began talking to members of Congress about the worm diseases like schistosomiasis and hookworm infection. By then, we were making vaccines for those conditions and for river blindness (lymphatic filariasis). Together with two English colleagues (Professors David Molyneux and Alan Fenwick), we were able to get legislation passed to support “neglected tropical diseases” in the US and UK. And that’s when I started getting my chops to do the public engagement and policy work and I found it very meaningful, even though it was a struggle to keep up a lab and grants at the same time.

*JCI*: In 2011, you moved to Baylor College of Medicine to start the National School of Tropical Medicine and the Center for Vaccine Development. What prompted that move?

Hotez: I loved being at Rockefeller and Cornell. I loved being at Mass General, at Yale, and I loved being at GW. I got a lot out of those institutions, but I wanted a bigger footprint. I saw the diseases I was interested in emerging in the US gulf coast. I came to Texas to up my game for science technology because of the Texas Medical Center — the home of Texas Children’s Hospital and Baylor College of Medicine — in Houston. It is such a city of innovation. We’ve since expanded the technology from the hookworm vaccine to other parasitic disease and viral vaccines; we began making coronavirus vaccines for SARS and MERS and ultimately turned that approach to COVID-19 to make COVID-19 vaccine prototypes that have reached 100 million people in low- and middle-income countries. That was always part of my plan. I always saw vaccines and vaccinology and being a pediatrician-scientist as one of the highest expressions of science for humanity.

Beyond that, there is another side to Texas, which I was able to use in public engagement because Texas had become the center of the antivaccine and the “medical freedom” movements in the US. By then, I had four adult kids, including my daughter Rachel, who has autism and intellectual disabilities. I — and most of the world outside of those movements — knew that there was no link between vaccines and autism, so I wrote the book *Vaccines Did Not Cause Rachel’s Autism* while I was in Texas, and that quickly made me public enemy number one with antivaccine groups. I saw this a learning opportunity because it gave me a front row seat to what the antivaccine movement was all about. Tragically, I’ve watched this only grow during the most recent pandemic. It was important to write the book about Rachel to take the wind out of their sails.

It has been hard on that front in Texas in this COVID-19 pandemic. So many Texans became victims of antiscience aggression coming from the political right, and ultimately 40,000 Texans (and 200,000 nationwide) needlessly died because they refused the SARS-CoV-2 vaccine. These were people that I knew, and particularly in the more conservative areas like in East Texas, Central Texas, or the Texas Panhandle, it was heartbreaking to watch. I got angry because I saw these people as real, incredibly warm, kind individuals who were victims. Victims of targeted political aggression from the far right — from members of Congress and some governors, all amplified on Fox News. That’s why I wrote my latest book (*The Deadly Rise of Anti-Science*), in their memory.

*JCI*: You are extremely sympathetic to people who come at you with antivaccine views, understanding that they might have been indoctrinated by false information or looking for a reason for their child’s malady.

Hotez: We use the terms misinformation or infodemic; I don’t use those terms because it implies it’s some random junk on the internet. It’s not: it’s organized. It’s deliberate. It’s predatory. There are predators from the antivaccine movement who are either monetizing the internet or using these individuals for their political gain. In this latest version, the antivaccine industry or movement has evolved into a political ecosystem, which in many cases is linked to extremist politics, and one that targets victims living in conservative counties or states. It’s very unpleasant to talk about; in our training as physicians and scientists we are taught not to talk about Republicans versus Democrats. We’re supposed to be politically neutral, and I get that. But what do you do when the data show overwhelmingly those who lost their lives were in red states, and the redder the county (red being Republican), the lower the immunization rate and higher the COVID death rate? It’s now a killing societal force, and I trained to become a pediatric vaccine scientist to save lives. I try to save lives through making vaccines, and now through combating this kind of antivaccine aggression because who else is going to do it? It’s not that we take an interest in people’s political leanings — that’s their fundamental right as American citizens, but rather we need to find ways to uncouple them from antiscience viewpoints because this has led to so many unnecessary American deaths. And now it’s globalizing. For me this been a tough struggle, but it’s also meaningful.

*JCI*: I recall you noting that tropical infections have less to do with weather than economics. Poverty is the common thread, and you went on to talk about the lack of funding for studying these pathogens and for developing associated vaccines. Do you think that will change now that we are mid/post SARS-CoV-2 pandemic?

Hotez: I don’t know. You’d think it would be wise to increase funding on research of not just neglected tropical diseases, but also into pandemic threats in general. I am not optimistic about seeing big changes, in part because of the antivaccine movement. What we are seeing now is a lot of revisionist history coming from the far right. Rather than having some self-reflection that the far right had a role in precipitating the deaths of 200,000 Americans by convincing them not to get a vaccine — instead what you’re seeing is an attempt to revise history and exhortations that it was the vaccines that killed Americans. We have solid scientific evidence that’s not the case, but that’s the narrative they’re putting out there, and even worse, they have put out the claim that it’s virologists who created the virus because of either purposeful gain-of-function mutations or lab leak. Yet we have six or seven published papers on the natural origins of SARS-CoV-2 in top journals versus none for the other assertions.

So I worry that this is going to be used as an excuse to diminish or degrade infectious diseases research in the US and maybe globally. The opportunities to make vaccines for both neglected diseases and emerging pandemic threats is unprecedented. The problem is not going to be the technology; instead it’s the politics in this new age of antiscience.

*JCI*: If you could not be a physician or a scientist, what other career would you choose?

Hotez: I really like being a professor, and being a medical school professor is exactly what I want to be doing. I also love writing books — so establishing myself as an author or writer has become an important activity for me. But being a professor seems to be in my DNA and RNA and translated proteins as well.

## Figures and Tables

**Figure 1:**
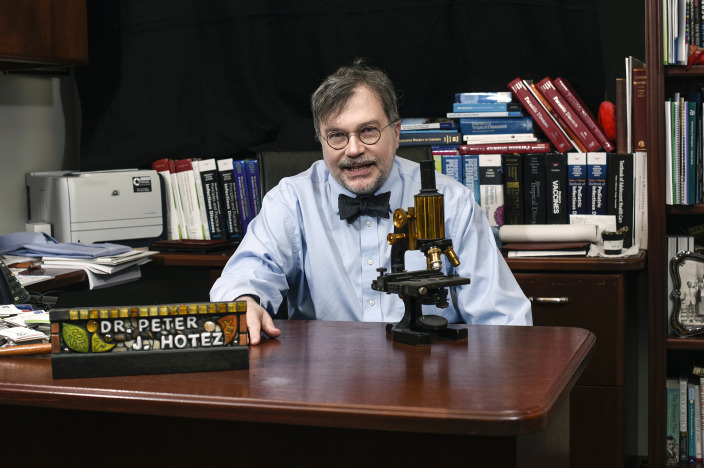
Peter Hotez. Image credit: Agapito Sanchez.

## Version 1 11/01/2023

Electronic publication

